# On the Origin of Hemoglobin Cooperativity under Non-equilibrium Conditions

**DOI:** 10.15190/d.2022.5

**Published:** 2022-06-30

**Authors:** Rosella Scrima, Sabino Fugetto, Nazzareno Capitanio, Domenico L. Gatti

**Affiliations:** ^1^Department of Clinical and Experimental Medicine, University of Foggia, Via L. Pinto 1, Foggia, Italy; ^3^Department of Biochemistry, Microbiology and Immunology, Wayne State University School of Medicine, 540 E. Canfield Avenue, Detroit, MI, USA

**Keywords:** Hemoglobin, mitochondria, kinetics, models, cooperativity.

## Abstract

Abnormal hemoglobins can have major consequences for tissue delivery of oxygen. Correct diagnosis of hemoglobinopathies with altered oxygen affinity requires a determination of hemoglobin oxygen dissociation curve, which relates the hemoglobin oxygen saturation to the partial pressure of oxygen in the blood. Determination of the oxygen dissociation curve of human hemoglobin is typically carried out under conditions in which hemoglobin is in equilibrium with O2 at each partial pressure. However, in the human body due to the fast transit of red blood cells through tissues hemoglobin oxygen exchanges occur under non-equilibrium conditions. We describe the determination of non-equilibrium oxygen dissociation curve and show that under these conditions the true nature of hemoglobin cooperativity is revealed as emerging solely from the consecutive binding of oxygen to each one of the four subunits of hemoglobin until the entire tetramer is saturated. We call this form of cooperativity the sequential cooperativity of hemoglobin and define the simplest model that includes it as the minimalist model of hemoglobin. A single instantiation of this model accounts for ~70% of hemoglobin cooperativity under non-equilibrium conditions. The total cooperativity of hemoglobin can be viewed more correctly as the summation of two instantiations of the minimalist model (each one corresponding to a tetramer of low and high affinity for O2, respectively) in equilibrium with each other, as in the Monod-Wyman-Changeux model of hemoglobin. In addition to offering new insights on the nature of hemoglobin reaction with oxygen, the methodology described here for the determination of hemoglobin non-equilibrium oxygen dissociation curve provides a simple, fast, low-cost alternative to complex spectrophotometric methods, which is expected to be particularly valuable in regions where hemoglobinopathies are a significant public health problem, but where highly specialized laboratories capable of determining a traditional oxygen dissociation curve are not easily accessible.

## INTRODUCTION

Hemoglobin (Hb) oxygen dissociation curve (ODC), which relates oxygen saturation (S_O2_) and partial pressure of oxygen in the blood (P_O2_), is an important tool for understanding how blood carries and releases oxygen^1^.

Classically, factors recognized to influence the ODC include the local CO_2_ partial pressure (P_CO2_), pH, temperature, as well as allosteric metabolites like 2,3 diphosphoglycerate (2,3-DPG). The curve is shifted to the right (i.e. lower saturation for a given P_O2_) by higher P_CO2_, greater acidity (lower pH), higher temperature, and higher concentration of 2,3-DPG^1-7^. The factors that shift the ODC to the right are directly relevant to the conditions that prevail in metabolizing tissues, as they facilitate the unloading of oxygen from hemoglobin. The converse occurs during passage through the pulmonary capillaries, with the greater affinity accompanying a shift of the ODC to the left aiding the uptake of oxygen^8^.

The partial pressure of oxygen in the blood at which hemoglobin is 50% saturated is known as the P_50_. The P_50_ of normal hemoglobin is approximately 26 mmHg at a partial CO_2_ pressure of 40 mmHg^7^. In the presence of disease or other conditions that change hemoglobin oxygen affinity and, consequently, shift the curve to the right or left, the P_50_ changes accordingly^^9^^. Low affinity hemoglobins are characterized by higher P_50_, and high-affinity hemoglobins by a lower P_50_. Such abnormal hemoglobins can have major consequences for tissue delivery of oxygen, but their effects are mitigated by various compensatory mechanisms, one of which is the hemoglobin concentration. High-affinity molecules, by definition, release oxygen less readily than normal and, because tissue hypoxia is a stimulus to hemoglobin production, affected individuals often have polycythemia; approximately 100 hemoglobin variants with high oxygen affinity have been described in the literature^10^. By contrast individuals with low affinity hemoglobins are usually anemic. For example, the abnormal hemoglobin (HbS) of sickle-cell disease (SCD) has reduced oxygen affinity. SCD is a monogenic disorder where the patient is a heterozygous or homozygous carrier for the b^S^ allele. This mutation causes a single amino acid substitution from glutamic acid to valine in the b-globin chain of Hb, which results in hydrophobic interactions with adjacent HbS molecules leading to HbS polymers. This polymerization process is triggered at low P_O2_ and is also dependent on pH. Early recognition in life of the presence of HbS in the blood erythrocytes can better prepare the patient for an SCD ‘event’ in which rigid, sickled erythrocytes adhere to the endothelium and cause painful vaso-occlusion crises (VOC).

Neonatal cyanosis can also be due to Hb variants with reduced oxygen affinity and a pronounced shift of the ODC to the right. With the introduction of universal screening for congenital heart disease, the finding of abnormal ODC’s will likely uncover more neonates with hemoglobinopathies with low oxygen affinity.

Finally, measurement of a complete ODC is important for detecting hemoglobin variants that may escape conventional tests measuring only P_50^10-12^_. For example, some abnormal ODC’s are characterized by bi-phasism due to the coexistence of both normal and high affinity hemoglobins, and to an exchange of subunits between them^12,13^. In these cases, measurement of the complete ODC is important to infer the functional properties of the individual hemoglobin components that cannot be easily separated.

Hemoglobin ODC was originally determined by manual methods in which the oxygen saturation of hemoglobin was measured by spectrophotometry at every stepwise change of P_O2_ by the addition of aliquots of air to the pre-deoxygenated sample in a tonometer, after equilibrium between Hb and O_2_ at each P_O2_ was reached. Although very accurate, this *static *method was very laborious and time consuming. To overcome its limitations, *dynamic* methods were developed in which P_O2_ was progressively changed in a close vessel, allowing for equilibration in between changes^1,14-18^. In the most current implementation of these methods, hemoglobin oxygenation and deoxygenation are achieved with highly purified supplies of oxygen and nitrogen or argon gases from cylinders, a Clark oxygen electrode detects the change in oxygen tension, while the resulting increase in oxyhemoglobin fraction is simultaneously monitored by dual-wavelength spectrophotometry at 560 nm and 576 nm^19^.

However, equilibrium ODC does not reflect correctly physiological gas exchanges by Hb in the lungs (oxygenation) and in peripheral tissues (deoxygenation) as, due to the fast transit of red blood cells (RBCs) through microcirculation^20^, hemoglobin O_2_ exchanges occur under non-equilibrium conditions. Here, we describe a simple method for the determination of non-equilibrium ODC, and analyze the contribution of different components of Hb cooperativity to the curve. Besides mimicking more closely physiological gas exchanges, the methodology offers a low-cost, low-tech alternative to complex and expensive commercial instruments for the determination of equilibrium ODC, which will undoubtedly be valuable in areas of the world where hemoglobinopathies represent a significant public health problem, and highly specialized laboratories capable of determining a traditional ODC are not easily or timely accessible.

## MATERIALS AND METHODS

Rat liver mitochondria were purified by differential centrifugation of tissue homogenate as described in^21^, portioned in aliquots of 30-40 mg protein/ml in 0.25 mM sucrose, and stored at -80 °C till used. *Stripped *Hb devoid of allosteric heterotropic factors was prepared from whole human blood of healthy donors^22^ and its concentration, as HbO_2_, was estimated spectrophotometrically from its heme content, with an ε_mM _at 577 nm of 15.4^23^. Alternatively, Vacutainer (Becton, Dickinson and company) collected whole blood was used; in this case the HbO_2 _concentration was estimated, after dilution of the sample in double distilled water, from the air-equilibrated minus Na_2_S_2_O_4_-supplemented differential spectra, using a Δε_mM_ at 577-568 nm of 4.8 according to^24^; the Hb content (as heme centers) was typically 9-11 nmol/(ml blood).

Determination of oxygen consumption was carried out by respirometry using Oxygraph-2k (O2k, OROBOROS Instruments, Innsbruck, Austria). The instrument has two measuring chambers (2 ml each) both equipped with a Clark-type electrode; calibration of the instrument was performed according to the manufacturer instructions and all the measurements were carried out at 37 °C. The assay medium constituted by 250 mM sucrose, 1 mg/ml bovine serum albumin, 10 mM KH_2_(PO_4_), 27 mM KCl, 1 mM MgCl_2_, 40 mM Hepes, 0.5 mM EGTA (pH 7.4) was supplemented with 20-50 mg/ml mitochondrial proteins in the absence or presence of either purified Hb or whole blood samples. Oxygen consumption was initiated by the addition of 10 mM succinate as respiratory substrate in the presence of 2 µM rotenone, an inhibitor of the respiratory chain NADH-ubiquinone oxidoreductase/Complex I. Alternatively, the measurements were carried out with a single-chamber (0.5-1 ml) oxymeter equipped with a Clark type electrode disc (Hansatech Instruments Ltd, King's Lynn, UK).

Fitting of the deoxygenation/reoxygenation curves of Hb using four different kinetic models (minimalist, Adair, Perutz, MWC) was carried out using in-house code written for Matlab® (deposited at https://github.com/dgattiwsu/HB_ODC). Using these models, a single set of rate constants was derived by global analysis of both the deoxygenation and reoxygenation traces.

## RESULTS

### Non-equilibrium ODC of human hemoglobin

In the following we describe a typical experimental determination of human hemoglobin non-equilibrium ODC.

2 ml of the assay buffer solution (pH 7.4) were placed inside each of the two glass chambers of the Oroboros oxymeter and supplemented with a small amount of purified rat liver mitochondria to a final concentration of 30 mg prot/ml. With the cell open to the environment, the solutions were allowed to equilibrate under stirring with atmospheric O_2_ until the observed O_2_ concentration (based on an earlier calibration of the electrode^25-27^) remained stable (~183 µM) for a few minutes.

Upon insulating the glass cells from air with glass stoppers (containing a small port for microsyringe additions) progressive reduction of the P_O2_ in the cell was achieved by adding 10 mM succinate according to the reaction catalyzed by the succinate oxidase segment of the respiratory chain (complexes II+III+IV: succinate dehydrogenase, ubiquinol:cytochrome c reductase, cytochrome c oxidase, respectively):

succinate + 1/2 O_2_ --> fumarate + H_2_O

**Figure 1:**
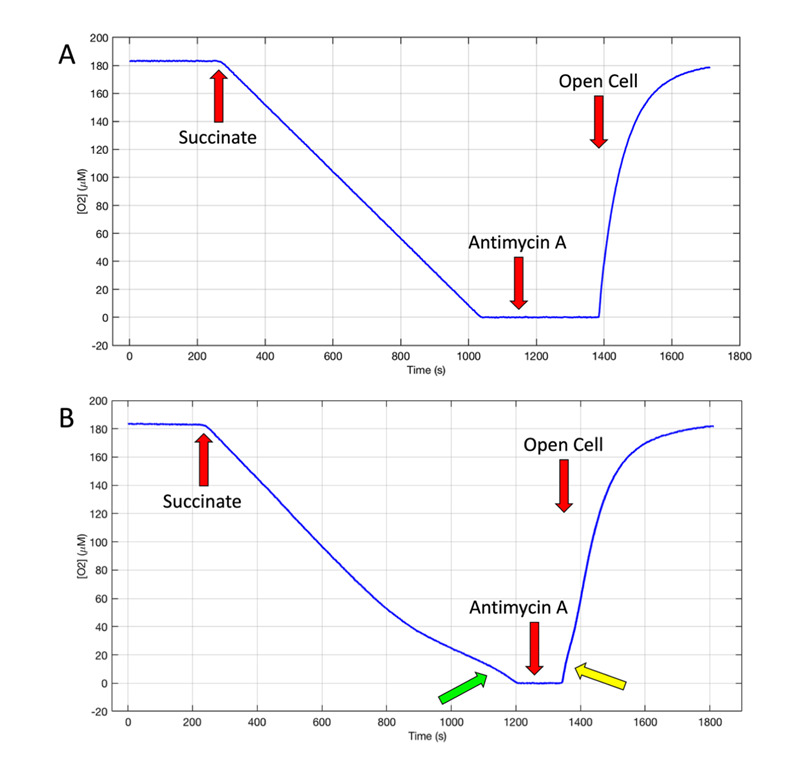
Experimental determination of Hb non-equilibrium ODC A. Polarographic trace of deoxygenation triggered by succinate, and reoxygenation triggered by reopening the Clark cell in the presence of Antimycin A. B. Deoxygenation / reoxygenation cycle in the presence of 50 mM Hb. A green and a yellow arrow point to the deviations from the pattern seen in A.

The final concentration of *cytochrome c oxidase* (the mitochondrial enzyme that uses up O_2_ reducing it to water) in the cell was estimated to be ~0.05 µ M by visible spectroscopy of the mitochondrial suspension in the 500-650 nanometer range. Upon starting respiration, O_2_ concentration decreased linearly until anaerobiosis was reached (Figure 1A).

The linear respiratory activity is due to the affinity of cytochrome *c* oxidase for O_2_ whose K_M_ is estimated in the sub-micromolar range^28,29^. Considering that the instrument limit of detection of O_2_ concentration is also in the submicromolar range^30^, the O_2_ concentration is practically never limiting the respiratory flux under the prevailing conditions reported here. After adding 5 mM Antimycin A, an inhibitor of respiration at the level of *ubiquinol:cytochrome c reductase*, the glass stopper was removed, and the cell content was allowed to equilibrate again with air. During this phase oxygen diffuses back in the cell in a non-linear fashion.

When the cycle of deoxygenation/reoxygenation is repeated in the presence of 50 mM (as heme centers) human hemoglobin isolated from blood hydrolysate (Figure 1B) the final part of the deoxygenation curve is no longer linear due to the release of oxygen from Hb (green arrow). The initial part of the reoxygenation curve is also slower due to the uptake of oxygen by Hb (yellow arrow).

**Figure 2:**
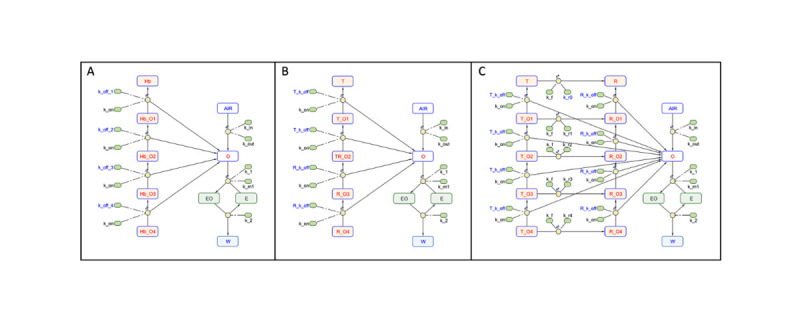
Kinetic models of Hb non-equilibrium ODC A. Adair model. B. Perutz model. C. MWC model. Species are shown as rounded rectangles: E = cytochrome c oxidase; O = cell O_2_; AIR = air O_2_; EO = cytochrome c oxidase:O_2_; W = water. Reactions are shown as yellow circles, rate constants as small, rounded rectangles. Rate constants that are refined in the model are highlighted in blue font. A double arrow over each reaction circle indicates full reversibility. Initial values for all the on rate constants were set at 100 mM^-1^s^-1^; initial values for the off rate constants were set accordingly based on the values of the equilibrium constants for the progressive binding of O_2_ to Hb as derived from an initial fit of the oximetric traces with a simple graphic method (Supplementary Information) and using Adair equation^1,16,33,34^. Initial value for the k_cat_ of cytochrome c oxidase were derived from the linear phase of respiration supported by succinate. In the Perutz model the species TR_O2 is used to represent the fast conformational transition from T_O2 to R_O2.

Since the alterations of the deox-/reoxygenation curves are due to the release/uptake of O_2 _by Hb, it is possible to recover the non-equilibrium ODC using a kinetic model of the cell ensemble as a set of reversible reactions involving the species of Hb in different oxygenation state, *cytochrome c oxidase *as the terminal O_2_ acceptor, O_2_ in the cell, and external air in diffusive equilibrium with the O_2_ in the cell, when the latter is open. We have evaluated three different kinetic models (Figure 2): **A**. a *sequential* Adair style model with 4 refined parameters (one O_2 _*k*_off_ for each Hb(O_2_)_n_), **B**. a *two state sequential* Perutz style model with 2 refined parameters (one O_2 _*k*_off_ for Hb(O_2_)_1,2 _and one O_2 _*k*_off_ for Hb(O_2_)_3,4_), and **C**. a *two state concerted* Monod-Wyman-Changeux (MWC) style model with 3 refined parameters (one *K*_equil_ between a *tense* (T) and a *relaxed* (R) state of Hb, one O_2 _*k*_off_ for all Tstatesand one O_2 _*k*_off_ for all R states) (reviewed in^31,32^).

**Figure 3:**
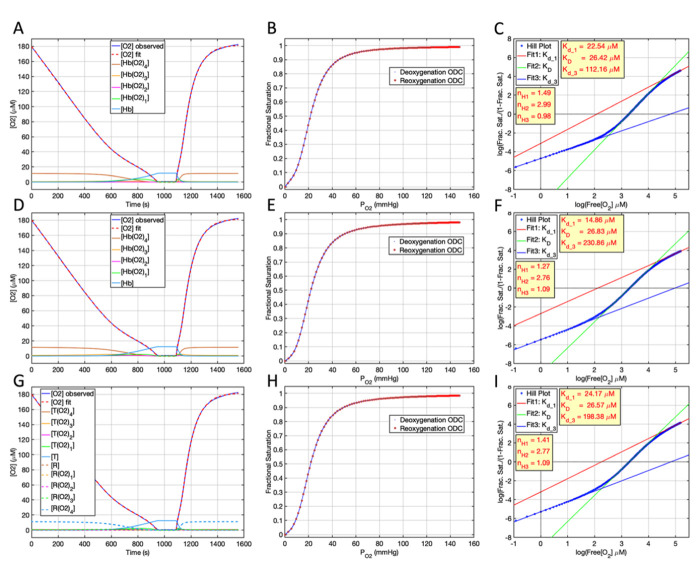
Hb non-equilibrium ODC with the Adair, Peruts, and MWC models A, D, G. Kinetic fit of the deoxygenation/reoxygenation cycle shown in Figure 1B using the Adair, Perutz, and MWC model, respectively. The refined concentration of Hb tetramers was 11.7, 12.4, 12.5 mM in the Adair, Perutz, and MWC models, respectively. B, E, H. ODC’s calculated from the fits in A, D, G, respectively, using only the deoxygenation (blue dots) or the reoxygenation (cyan circles with red outline) phase. The concentrations of the different Hb species refer to the entire tetramer: therefore, fractional saturation is calculated as ([Hb(O_2_)_4_] x 4 + [Hb(O_2_)_3_] x 3 + [Hb(O_2_)_2_] x 2 + [Hb(O_2_)])/([Hb_total_] x 4). The calculated P_50_ from a non-linear least-squares fit of all the points in the ODC’s with Adair equation is ~21.9 mmHg. C, F, I. Corresponding Hill plots calculated combining points from the deoxygenation and reoxygenation phase.

The three models fit equally well the oxymetric trace with essentially identical *sum of square errors *(~150 µM^2^) and *R-square* values (>0.99). Each model provides the contribution of all Hb species (Figure 3A,D,G) at all time points (O_2_ concentrations). Non-equilibrium ODC’s are derived for each P_O2_ value as the ratio between the sum of all the oxygenated species and the total amount of Hb (Figure 3B,E,H). The corresponding Hill plots are shown in Figure 3C,F,I.

**Figure 4:**
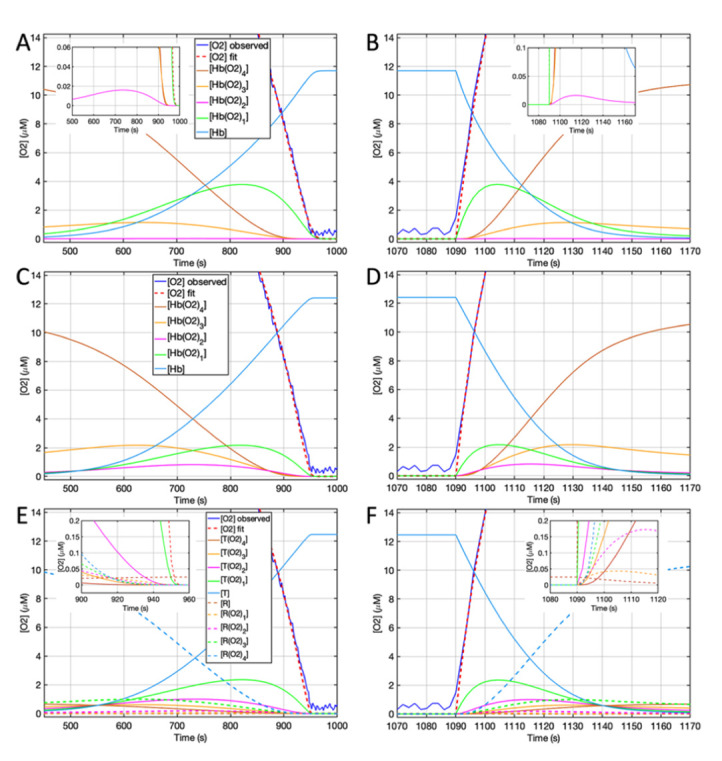
Kinetic fit of the deoxygenation/reoxygenation cycle shown in Figure 1B using the Adair, Perutz, and MWC model, respectively A,C,E. Blow-up of the terminal part of Hb deoxygenation phase. B,D,F. Blow-up of the initial part of Hb reoxygenation phase. Insets in A,B show the peaks of Hb(O_2_)_2_. Notice the cross-over points at 520 s (C) and 1146 s (D) between Hb, Hb(O_2_)_1_, Hb(O_2_)_2_, corresponding to Perutz T conformation, and at 870 s (C) and 1098 s (D) between Hb(O_2_)_2_, Hb(O_2_)_3_, and Hb(O_2_)_4_, corresponding to Perutz R conformation. Likewise, cross-over points are present at 917 s (E, inset) and 1093 s (F, inset) between all R states, and at 551 s (E) and 1040 s (F) between all T states of the MWC model.

The observed variations in the ODC and Hill plots are due to the fact that for each time point the contributions of individual Hb species are different in the three models. Details of these contributions are shown in a blow-up of the terminal part of Hb deoxygenation phase (Figure 4A,C,E), and of the initial part of Hb reoxygenation phase (Figure 4B,D,F). Regardless of the model used, concentration peaks are reached in the order Hb(O_2_)_4_ --> Hb(O_2_)_3_ --> Hb(O_2_)_2_ --> Hb(O_2_)_1_ --> Hb during deoxygenation, and Hb --> Hb(O_2_)_1_ --> Hb(O_2_)_2_ --> Hb(O_2_)_3_ --> Hb(O_2_)_4_ during reoxygenation.

In the Perutz and MWC models, intercepts with the X axis of the extrapolated lines from the asymptotic ends of the Hill plot are usually interpreted as the concentration of O_2_ at which the concentrations of the unliganded and liganded forms of the T and R states are equal (*K*_D_). Better values for these magnitudes are calculated directly from the cross-over points (Figure 4) of the T and R forms derived from the kinetic simulations (Table 1). Non-linear least-squares fit of Adair equation^16,33,35^,

S_O2_ = K_1_[O_2_]+2K_1_K_2_[O_2_]^2^+3K_1_K_2_K_3_[O_2_]^3^+4K_1_K_2_K_3_K_4_[O_2_]^4^ / 4(1+K_1_[O_2_]+2K_1_K_2_[O_2_]^2^+3K_1_K_2_K_3_[O_2_]^3^+4K_1_K_2_K_3_K_4_[O_2_]^4^)

K_1_ = [Hb(O_2_)] / [Hb][O_2_]; K_2_ = [Hb(O_2_)_2_] / [Hb(O_2_)][O_2_]; K_3_ = [Hb(O_2_)_3_] / [Hb(O_2_)_2_][O_2_]; K_4_ = [Hb(O_2_)_4_] / [Hb(O_2_)_3_][O_2_];

**Table 1:** KD’s (µM) from model refinement, and Adair equilibrium (association) constants (µM-1) for the four steps of Hb oxygenation from a fit of Adair equation to the non-equilibrium ODC’s For the Adair and Perutz model, these values are identical to those refined during the optimization of the two models. Notice the collapse of 4 Adair constants into just 2 in the Perutz model, and just 1 in the Minimalist model (see below, Sources of cooperativity in the models). In the MWC model, K_T_ and K_R_ are the refined association constants for the T and R states oxygenation, c = K_T_/K_R_, and L = [T]/[R] is the refined allosteric constant expressing the equilibrium in the absence of any oxygenation between the T and R state of Hb. Allosteric constants L_n_ for the n different oxygenation levels are not refined, but calculated according to the relationship L_n_ = Lc^n^.

	Imai^1^	Minimalist model	Adair model	Perutz model	MWC model c = 0.1069, L0=504.6, L1=53.9, L2= 5.76, L3= 0.62, L4= 0.066, KT = 0.0191, KR = 0.1787
KD_crossover		27.95 (=1/K1)			
KD_Hill		27.73	26.42	26.83	26.57
KDT_crossover				59.68 (=1/K1)	52.09 (=1/KT)
KDR_crossover				12.04 (=1/K4)	5.69 (=1/KR)
K1	0.0188	0.0360	0.0368	0.0168	0.0194
K2	0.0566	0.0360	0.0002	0.0168	0.0220
K3	0.4070	0.0360	2.2237	0.0818	0.0428
K4	4.2800	0.0360	0.1730	0.0818	0.1178

to the combined experimental points from the deoxygenation and reoxygenation *non-equilibrium* ODC’s can be used to derive values for the *equilibrium* Adair constants in the three models (Table 1)**. **

### Sources of cooperativity in the models

**Figure 5:**
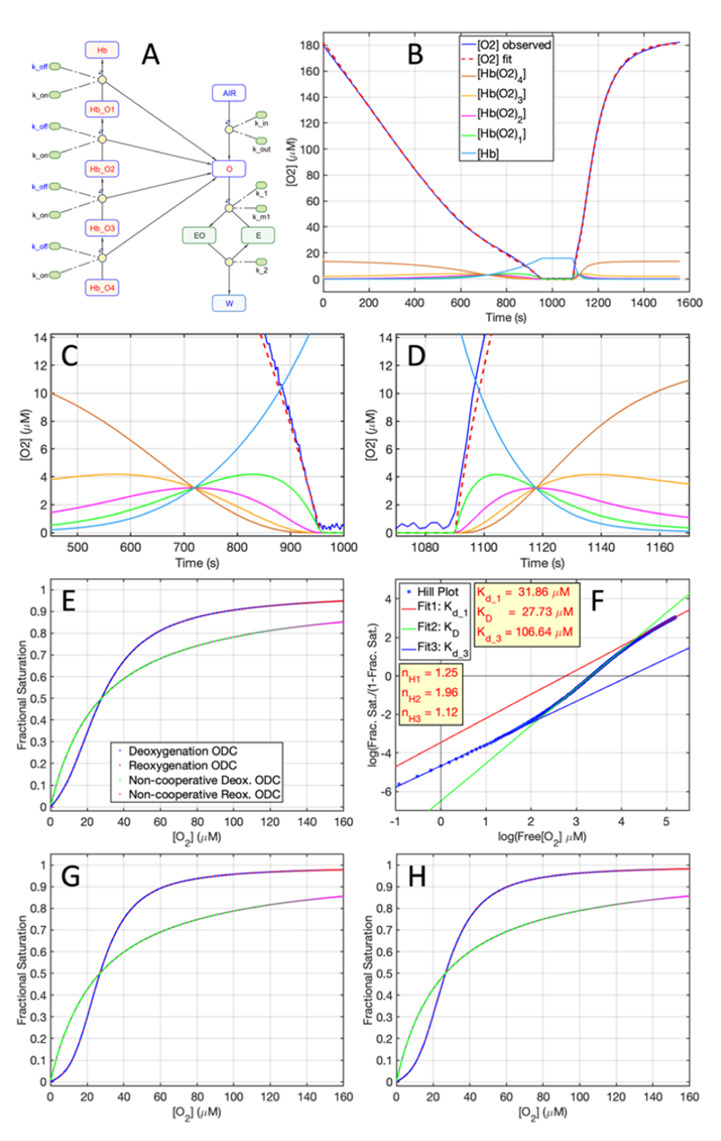
Hb non-equilibrium ODC with the minimalist model A, Minimalist model with the same O_2_ affinity at all binding sites. B, Kinetic fit of the deox/reox cycle shown in Figure 1B. C, D. Blow-up of the terminal part of Hb deoxygenation phase and the initial part of Hb reoxygenation phase. E, ODC calculated from the fit in panel B, using only the deoxygenation (blue dots) or the reoxygenation (red circles) phase. The ODC with the same P_50 _derived from a model containing 4 independent identical O_2_ sites is shown for the same phases as green and magenta dots (cgain = 0.1052). F,Corresponding Hill plot calculated combining points from the deoxygenation and reoxygenation phase. G, H, ODC’s showing the cooperativity gain of the Perutz (cgain = 0.1505) and MWC models (cgain = 0.1526).

A common feature of Adair, Perutz, and MWC models is the presence of sequential reactions. A *minimalist *model containing four sequential binding reactions, Hb <--> Hb(O_2_)_1_ <--> Hb(O_2_)_2_ <--> Hb(O_2_)_3_ <--> Hb(O_2_)_4_, with a single O_2_ affinity for all states, and no conformational changes (Figure 5A), fits experimental observations surprisingly well (*sse* = 515.5, *R^2^* = 0.998) (Figure 5B,C,D), giving origin to a sigmoidal ODC (Figure 5E) and a Hill plot with asymptotic components that suggest the presence of both low and high affinity sites (Figure 5C), despite none such exist in the model. Accordingly, a fit of Adair equation to the sigmoidal ODC shows that all four Adair constants collapse into a single one corresponding to the refined *k_on_/k_off_* ratio (Table 1). This observation suggests that the most basic source of cooperativity in the Adair, Perutz, and MWC models resides in the fact that all three models feature *sequential *binding reaction of O_2_ to the four sites of Hb.

We define as the *cooperativity gain *(*cgain*) of a model the *rms* (root mean square) deviation between the model derived ODC and the ODC with the same P_50 _derived from a model containing 4 independent identical O_2_ sites. The *cooperative gain *of the minimalist, Perutz and MWC models is shown in Figure 5E,G,H.

## CONCLUSION

We have presented a typical experiment showing the determination of hemoglobin non-equilibrium ODC, and from it the determination of key parameters such as the P_50_ and the Hill’s coefficient. The experimental component of the method requires minimally the acquisition of a deoxygenation curve of Hb and, optionally, also that of a reoxygenation curve. The computational component is based on the minimization of the *sum of square errors *(*sse*) between an experimentally observed O_2_ polarographic trace and a simulated O_2 _trace based on a kinetic model of choice (Figures 2-4). Since it does not require an optical determination of the Hb saturation, this method can be directly used with a red cell suspension or whole blood without the added complications of the dual-wavelength or full sphere spectrometry that are necessary to eliminate light scattering noise^14^. Dedicated instruments capable of carrying out the determination of hemoglobin equilibrium ODC are commercially available at a significant cost, as they require supplies of highly purified gases (i.e., O_2_, N_2_, Ar) from cylinders, a Clark-type electrode to measure hematology laboratories of large medical centers. On the contrary, the protocol described here for the determination of non-equilibrium ODC requires only a widely available glass cell of non-optical quality equipped with a Clark-type electrode, and no flushing of the solutions with purified gases. In our experiment, we have used an in-house preparation of rat liver mitochondria as the source of the respiratory enzymes that drive anaerobiosis. However, a preparation of bacterial membrane particles with comparable properties is commercially available as EC-Oxyrase^®^. Finally, while in our study we have used the proprietary computational platform Matlab^®^ for model generation and for the analysis of experimental traces, our code will run also with few modifications in the open-source platform GNU-Octave, and it can be easily converted into fully-open source Python code. Thus, altogether, the methodology described here can be easily set up in a small laboratory of a neighborhood clinic as a low-cost, low-tech alternative to the expensive commercial instrumentation available only in large medical centers. A simple determination of Hb ODC can be very useful as part of an initial population screening for hemoglobinopathies before further molecular validation is sought, and is expected to be particularly valuable in regions where hemoglobinopathies are a significant public health problem, but highly specialized hematology laboratories are not easily accessible.

It has not escaped our notice that the chemical constants derived from *non-equilibrium* ODC are significantly different from those reported by other authors (i.e.^1^, Table 1), based on *equilibrium *ODC. This is not surprising, since *non-equilibrium* ODC mimics the physiological condition of red blood cells moving rapidly in the blood stream across regions of different P_O2_, while this condition is not reproduced in *equilibrium *ODC.

Three kinetic models (Adair *sequential*, Perutz *two state sequential*, MWC *two state concerted*) were all equally effective in fitting the experimental polarographic traces. Thus, as such, the determination of *non-equilibrium* ODC does not offer any new ways to discriminate between these models. However, the predictions made by the three models with respect to the concentrations of oxygenation and conformational intermediates are quite different (Figure 4) and may offer inspiration for future experiments.

The origin of Hb sigmoidal ODC has been a point of intense debate for over a century (reviewed in^31,32,34^), and this curve is perhaps the most frequently used ‘book perfect’ example of *positive cooperativity*. Our experiments were carried out with *stripped *Hb devoid of allosteric heterotropic factors, and thus under these conditions we observed the intrinsic cooperative behavior of tetrameric Hb. A *minimalist* model, containing no induced (by O_2_ binding) or intrinsic conformational equilibria between a *low* and a *high* affinity state, is sufficient to provide, by virtue of the constraint of *sequential *binding reactions of identical affinity, a large fraction (~69% in *cgain* scale) of the cooperative behavior of Hb, as judged by the magnitude of its *cooperative gain* (= 0.1052) with respect to a model containing 4 independent identical O_2_ binding sites. We call this basic level of cooperativity observed in the *minimalist *model, the *sequential cooperativity *of Hb. The constraints of sequential binding of oxygen molecules to Hb (rather than, for example, the simultaneous binding of two O_2_ molecules to distinct subunits of a single Hb molecule with formation of a single transition state) does not require the invocation of any special structural features of Hb, because according to collision theory the probability of three chemical species reacting simultaneously with each other in a dilute solution in a termolecular elementary reaction is negligible^36,37^. Therefore, apparent termolecular reactions are typically viewed as non-elementary reactions that can be broken down into a more fundamental set of bimolecular reactions in agreement with the law of mass action.

The *total* cooperativity of Hb under non-equilibrium conditions is accounted for by inclusion in the model of a single conformational equilibrium between a *low* and a *high* affinity state, as shown by the values of the *cooperative gain *in the Perutz (*cgain* = 0.1505) and MWC models (*cgain* = 0.1526). One particularly interesting way of explaining the conceptual relationship between the *sequential *and the *total* cooperativity of hemoglobin is to view *total *cooperativity as the summation of two or more realizations of *sequential *cooperativity. From this point of view, the constraint of sequential reactions is the only true requirement for the emergence of a cooperative behavior. For example, the MWC model (Figure 2C) can be viewed as the summation of two *minimalist* models (Figure 5A), each one with a different single O_2_ affinity for all states, in equilibrium with each other. In this respect, the Perutz model does not offer any additional insights on the origin of Hb cooperativity, and historically has been perhaps just a source of confusion on this issue.

The observation that Hb cooperativity might originate simply as the consequence of consecutive reactions of a single Hb molecule with four oxygen molecules, paves the way for the exploration of other enzymatic systems, in which phenomena of cooperativity have been attributed solely to conformational changes. At this point it is not clear whether the *sequential *nature of Hb cooperativity was not discovered previously simply because all earlier determinations of Hb ODC were carried out under equilibrium conditions. When a reversible reaction is observed under equilibrium conditions, the forward and backward rates are necessarily identical. However, earlier computational work has revealed that the path followed by the forward and reverse reactions can be different in enzyme conformational space^38,39^. The constraint of equality between forward and backward rates at equilibrium is more easily fulfilled if two distinct conformations of Hb can affect both rates. Instead, if Hb oxygenation occurs under non-equilibrium conditions, in the absence of a constraint of equality between forward and backward rates at each oxygenation step, hemoglobin may exist almost entirely in one conformational state.

A major contribution of this study to the understanding of the cooperative behavior of allosteric proteins is the indication that the kinetic parameters associated with conformational transitions might be affected by the measuring conditions. The amount of conformational change required to account for the microscopic forward and backward reaction rates of each oxygenation step may depend on the rate at which P_O2_ changes. On this basis, it is tempting to speculate that *in vivo* the relative contribution of the two *minimalist *components that make up the MWC model of Hb, may be modulated by the rate at which erythrocyte move through the capillary bed. In general, one can anticipate that factors that increase this rate (e.g., lower plasma viscosity, higher flexibility of the erythrocytes membrane and cytoskeleton) will magnify the non-equilibrium character of Hb oxygenation, and thus will increase the contribution of only one conformational state of Hb. Conversely, factors that decrease this rate (e.g., higher plasma viscosity, as it occurs in extreme exercise^40^, infection and/or inflammation^41^, or decreased erythrocytes flexibility, as it occurs in an SCD ‘crisis’) will magnify the equilibrium character of Hb oxygenation, and thus will increase the contribution of both conformational states of Hb.
